# Leaf Senescence Regulation Mechanism Based on Comparative Transcriptome Analysis in Foxtail Millet

**DOI:** 10.3390/ijms25073905

**Published:** 2024-03-31

**Authors:** Xiaoxi Zhen, Chao Liu, Yajun Guo, Zirui Yu, Yuanhuai Han, Bin Zhang, Yinpei Liang

**Affiliations:** College of Agriculture, Shanxi Agricultural University, Jinzhong 030801, China; xiaoxizhen@sxau.edu.cn (X.Z.); liuchao20220714@163.com (C.L.); guo_yj0122@163.com (Y.G.); 13289026069@163.com (Z.Y.); binzhang@sxau.edu.cn (B.Z.)

**Keywords:** foxtail millet, dark-induced senescence (DIS), physiological changes, transcriptome analysis, senescence-associated genes (*SAG*s)

## Abstract

Leaf senescence, a pivotal process in plants, directly influences both crop yield and nutritional quality. Foxtail millet (*Setaria italica*) is a C_4_ model crop renowned for its exceptional nutritional value and stress tolerance characteristics. However, there is a lack of research on the identification of senescence-associated genes (*SAG*s) and the underlying molecular regulatory mechanisms governing this process. In this study, a dark-induced senescence (DIS) experimental system was applied to investigate the extensive physiological and transcriptomic changes in two foxtail millet varieties with different degrees of leaf senescence. The physiological and biochemical indices revealed that the light senescence (LS) variety exhibited a delayed senescence phenotype, whereas the severe senescence (SS) variety exhibited an accelerated senescence phenotype. The most evident differences in gene expression profiles between these two varieties during DIS included photosynthesis, chlorophyll, and lipid metabolism. Comparative transcriptome analysis further revealed a significant up-regulation of genes related to polysaccharide and calcium ion binding, nitrogen utilization, defense response, and malate metabolism in LS. In contrast, the expression of genes associated with redox homeostasis, carbohydrate metabolism, lipid homeostasis, and hormone signaling was significantly altered in SS. Through WGCNA and RT-qPCR analyses, we identified three *SAG*s that exhibit potential negative regulation towards dark-induced leaf senescence in foxtail millet. This study establishes the foundation for a further comprehensive examination of the regulatory network governing leaf senescence and provides potential genetic resources for manipulating senescence in foxtail millet.

## 1. Introduction

Leaf senescence is the final stage of leaf development, characterized by an active and orderly process of degradation and recycling of intracellular components. The mesophyll cells undergo intricate metabolic and structural alterations, encompassing chlorophyll degradation, reduced photosynthetic efficacy, starch accumulation, breakdown of macromolecules such as lipids, proteins, and nucleic acids, and disintegration of organelles like chloroplasts [1,2].

The process of leaf senescence is regulated by internal developmental signals and intricate external environmental cues while also being influenced by the levels of sugar, calcium ions, reactive oxygen species, and hormones [3,4]. Endogenous factors encompass the developmental age of plant leaves and various plant hormones, whereas exogenous environmental factors primarily comprise biological elements such as pathogen infections and abiotic factors like light intensity, temperature fluctuations, drought conditions, and nutritional deficiencies [5,6,7]. Chlorophyll degradation and chloroplast disintegration serve as indicators for the initiation of leaf senescence, and numerous cloned genes associated with chloroplast development and chlorophyll degradation have been linked to the process of senescence. The process of chlorophyll catabolism is regulated by NONYELLOWING/Stay-Green (*NYE1/SGR1*), PHEOPHYTINASE (*PPH*), NONYELLOW COLORING1 (*NYC1*), and pheophorbide an oxygenase (*PAO*), ultimately resulting in the degradation of green pigments in senescent leaves [8,9]. 

Physiological and biochemical changes during leaf senescence are affected by the expression of *SAG*s. Transcription factors (TFs), particularly NAC and WRKY TFs, are pivotal in governing the regulation of *SAG* expression [2]. For instance, the Arabidopsis NAC transcription factor ORE1/NAC2 orchestrates the integration of endogenous and environmental cues by regulating the expression of multiple *SAG*s and also the degradation of nucleic acids and chlorophyll to accelerate leaf senescence [10,11]. ANAC087 exerts a positive regulatory role in leaf senescence by activating the expression of multiple genes associated with chlorophyll degradation, reactive oxygen species production, and programmed cell death in Arabidopsis [12]. WRKY57 directly inhibits *SAG* expression by interacting with key inhibitors JAZ4 and JAZ8 of the jasmonic acid (JA) signaling pathway and negatively regulates leaf senescence induced by JA [13]. WRKY71 directly activates the expression of genes related to ethylene synthesis and signaling pathways and positively regulates leaf senescence in *Arabidopsis thaliana* by promoting ethylene synthesis [14]. In addition to transcriptional regulation, senescence is also regulated by post-translational modifications, such as ubiquitination and phosphorylation [2]. The degradation of the AtGDS1 transcription factor by the ubiquitination pathway under the action of the APC/C E3 ubiquitin ligase complex relieved its inhibitory effect on the expression of downstream *SAG*s, resulting in premature leaf senescence in Arabidopsis [7]. Receptor-like kinases (RLKs) are important proteins for phosphorylation that play a vital role in the regulation of leaf senescence. Recent studies showed that the senescence-related RLK SENRK1 functioned downstream of the senescence-promoting transcription factor WRKY53 and negatively regulated age-dependent leaf senescence in Arabidopsis [15], whereas a G-type LecRLK with active kinase and autophosphorylation activities encoding gene *PWL1* positively regulated leaf senescence in rice [16]. 

Leaf senescence is also regulated by the crosstalk among hormones. For instance, the renowned phytohormone JA plays a crucial role in regulating leaf senescence by interacting with auxin, gibberellin, and ethylene [17]. Moreover, the cytokinin oxidase/dehydrogenase coding gene *OsCKX11* is the key gene of antagonistic regulation of cytokinin and abscisic acid on rice leaf senescence. Knocking out this gene effectively delays rice leaf senescence, enhances photosynthesis, and increases grain number [18]. The auxin response factor TaARF15-A1 plays a negative regulatory role in the biosynthesis of senescence-promoting hormones (abscisic acid, ethylene, and salicylic acid), while positively regulating the expression of anti-senescence-related genes to participate in the negative regulation of wheat leaf senescence [19]. 

Foxtail millet (*Setaria italica*) is an important C_4_ photosynthetic plant with prominent drought resistance and stress resistance [20]. Recently, the establishment of a graph-based pan-genome further promoted the foxtail millet into a C_4_ genetic model plant [21]. Leaf senescence has a significant effect on crop yield and quality, but only two genes related to leaf senescence regulation have been reported in foxtail millet. *SiNAC1*, an NAC transcription factor coding gene in foxtail millet, positively regulates the natural senescence and dark-induced senescence of Arabidopsis in an ABA-dependent manner [22]. *SiYGL2* participates in the regulation of leaf senescence in foxtail millet by affecting chlorophyll content and photosynthetic capacity [23]. However, the regulatory mechanism of leaf senescence in foxtail millet remains unresolved. Therefore, the discovery of novel *SAG*s in foxtail millet holds significant biological significance for comprehending its senescence regulatory network.

Transcriptomic analyses have identified a large number of the underlying *SAG*s and TFs in crops, including maize, sorghum, wheat, and rice, which provide valuable insights into the regulation of leaf senescence [24,25,26,27]. Dark treatment is a stable and controlled carbon-starvation-induced senescence system, extensively employed for the characterization and analysis of various senescence phenotypes [28,29]. Here, we systematically analyzed the leaf senescence progression of 360 accessions from the core collection of foxtail millet [30] by DIS at the seedling stage and screened extreme varieties exhibiting either LS or SS phenotypes. The phenotypic, physiological, and antioxidant enzyme activity characteristics of the two varieties were investigated. Additionally, we conducted a comparative transcriptome analysis between the two varieties under light or dark conditions to identify differentially expressed genes and biological processes associated with leaf senescence in foxtail millet. Furthermore, we screened for the key candidate genes that potentially regulate leaf senescence in foxtail millet. This study lays the foundation for an in-depth analysis of the leaf senescence regulation network and provides important gene resources for the molecular breeding for foxtail millet varieties with high and stable yield.

## 2. Results

### 2.1. Phenotypic and Physiological Responses of Foxtail Millet Seedlings to Dark-Induced Senescence

**Figure 1:**
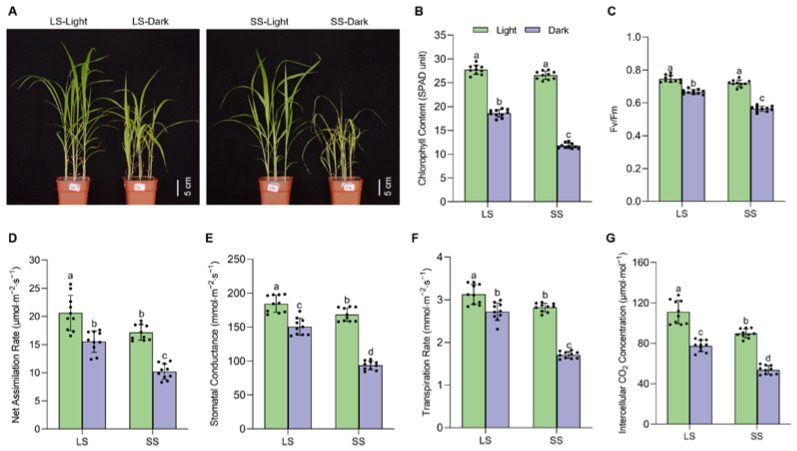
Differences in phenotype and photosynthetic parameters in two foxtail millet varieties during dark-induced senescence. (**A**) Representative images; (**B**) chlorophyll content (in SPAD unit); (**C**) Fv/Fm value; (**D**) net assimilation rate; (**E**) stomatal conductance; (**F**) transpiration rate; (**G**) intercellular CO_2_ concentration in 4-week-old seedlings leaves of LS and SS varieties under light or dark conditions for 7 d. Data are mean ± SD (*n* = 10 independent biological samples); different letters above the bars indicate statistically significant differences at *p* < 0.05 by one-way ANOVA, followed by Tukey’s post hoc tests for multiple comparisons.

We employed a DIS system to analyze the seedling senescence patterns of 360 foxtail millet accessions, and substantial phenotypic variations were discovered. To investigate the physiological responses of foxtail millet seedlings with varying degrees of senescence to DIS, the senescence-related traits in two representative varieties of LS (B56) and SS (B304) were determined at 7 days after light and dark treatment. Phenotypic analysis showed that the leaf yellowing of SS during DIS was significantly accelerated compared with LS (Figure 1A), as indicated by the different degrees of chlorophyll content reduction. Compared with LS, the chlorophyll content in SS showed a sharper decline during DIS (Figure 1B), and the maximum photochemical efficiency (Fv/Fm) of SS was also significantly reduced (Figure 1C). As reductions in chlorophyll may have an impact on photosynthetic capability, we further compared the photosynthetic gas exchange between LS and SS. The net assimilation rate (An), stomatal conductance (gs), intercellular CO_2_ concentration (Ci), and transpiration rate (E) in LS were significantly higher than those in SS under light conditions. Furthermore, the above photosynthetic parameters were reduced by DIS in both varieties, while the levels declined more sharply in SS than in LS (Figure 1D–G).

**Figure 2:**
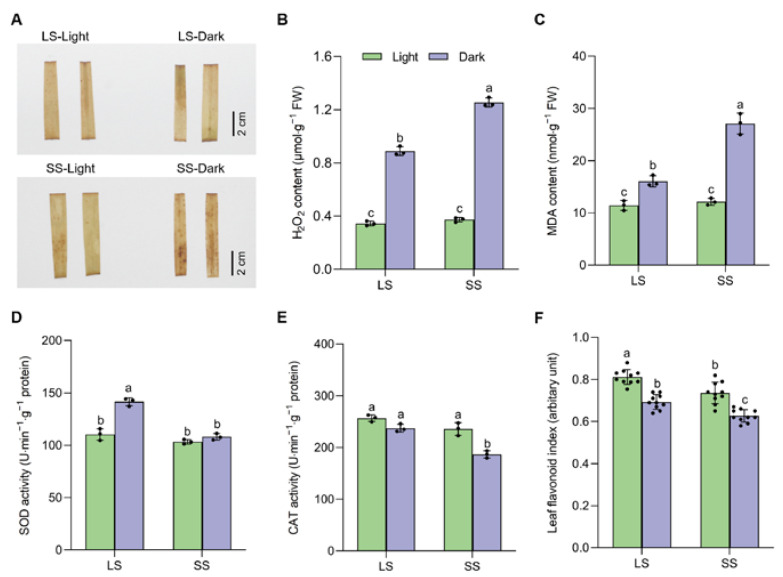
Differences in ROS accumulation and its enzymatic scavenging system in two foxtail millet varieties during dark-induced senescence. (**A**) DAB histochemical staining of leaves; (**B**) H_2_O_2_ content; (**C**) MDA content; (**D**) SOD activity; (**E**) CAT activity; (**F**) leaf flavonoid index in 4-week-old seedlings of LS and SS varieties under light or dark conditions for 7 d. Data are mean ± SD (*n* = 3 independent biological samples in A-E, *n* = 10 in F); different letters above the bars indicate statistically significant differences at *p* < 0.05 by one-way ANOVA, followed by Tukey’s post hoc tests for multiple comparisons.

Chlorophyll loss also causes reactive oxygen species (ROS) to accumulate. To evaluate the oxidative damage of foxtail millet seedlings during DIS, we examined the ROS accumulation by quantitative measurement of the hydrogen peroxide (H_2_O_2_) content and 3,3′-diaminobenzidine (DAB) staining. The H_2_O_2_ content was increased more in SS than in LS seedlings after dark treatment, with 1.56- and 2.32-fold increases, respectively. DAB staining also showed that the accumulation of H_2_O_2_ in SS was significantly more than that in LS (Figure 2A,B). Further investigation showed that the lipid peroxidation marker malondialdehyde (MDA) content was increased by DIS but with significantly higher accumulation in SS compared with LS (Figure 2C). Moreover, SS exhibited an apparently lower induction in superoxide dismutase (SOD) activity than LS after dark treatment, and the catalase (CAT) activity in SS was also lower (Figure 2D,E). Flavonoids have been reported to mitigate oxidative damage in plants. Compared to SS, LS exhibited a significantly higher leaf flavonoid index both under light and dark conditions (Figure 2F). 

**Figure 3:**
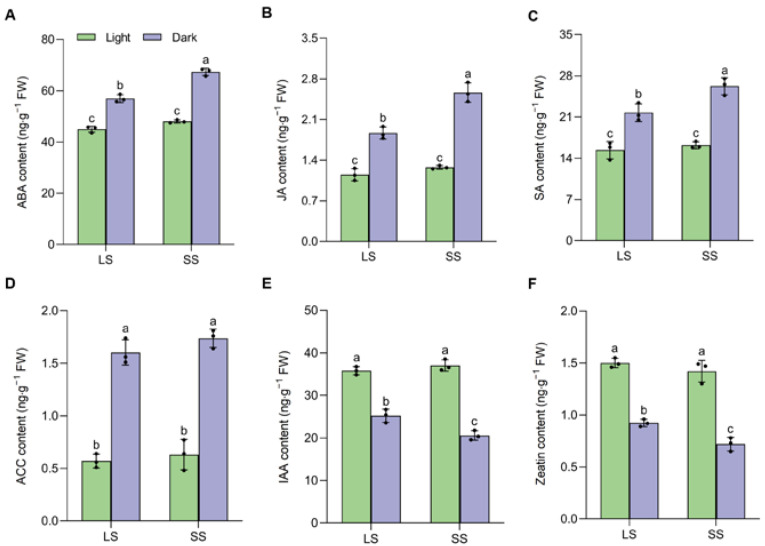
Differences in senescence-related phytohormone levels of two foxtail millet varieties during dark-induced senescence. (**A**) Abscisic acid (ABA) content; (**B**) jasmonic acid (JA) content; (**C**) salicylic acid (SA) content; (**D**) 1-Aminocyclopropane-1-carboxylate acid (ACC) content; (**E**) Indole-3-acetic acid (IAA) content; (**F**) zeatin content. Data are mean ± SD (*n* = 3 independent biological samples); different letters above the bars indicate statistically significant differences at *p* < 0.05 by one-way ANOVA, followed by Tukey’s post hoc tests for multiple comparisons.

Subsequently, we examined the levels of senescence-related phytohormones in both LS and SS seedlings. As shown in Figure 3, no significant differences were observed in the contents of all detected phytohormones between LS and SS seedlings under normal light conditions. Upon dark treatment, senescence-promoting phytohormones, including ABA, JA, SA, and ACC (ethylene), exhibited significant increases in both varieties, whereas senescence-delaying hormones like IAA (auxin) and zeatin (cytokinin) declined sharply. Nevertheless, the acceleration of senescence-promoting phytohormones levels was more pronounced in SS than in LS, excluding ACC (Figure 3A–D). Conversely, the contents of IAA and zeatin decreased more significantly in SS than in LS following dark treatment (Figure 3E,F).

### 2.2. DIS Elicited Substantial Transcriptomic Changes between LS and SS Foxtail Millet

**Figure 4:**
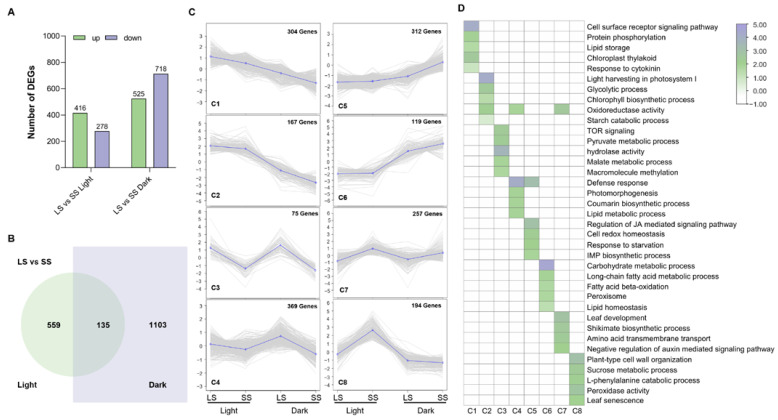
Clustering and functional analysis of DEGs in two foxtail millet varieties during dark-induced senescence. (**A**) The number of up- or down-regulated DEGs in the LS vs. SS group under light or dark conditions; (**B**) Venn diagram of DEGs in the LS vs. SS group under light and dark conditions; (**C**) cluster analysis and the average expression profiles of DEGs in two foxtail millet varieties under light or dark conditions; (**D**) gene ontology (GO) enrichment analysis of DEGs belonging to each cluster. Color bars indicate the degree of enrichment, presented by Log_10_ (*p*-values); *p*-value is the significance of the enriched GO terms in each cluster.

To investigate the transcriptional regulation of the foxtail millet seedlings in response to DIS, we conducted RNA sequencing to determine the transcriptome changes in LS and SS seedlings subjected to either light or dark conditions for 7 days. As shown in Figure 4A, we identified 694 differentially expressed genes (DEGs) in the light-grown seedlings of LS relative to those in SS, including 416 up-regulated and 278 down-regulated genes. However, there were 1238 transcripts significantly affected in LS seedlings grown in the dark treatment relative to the SS, including 525 up-regulated and 718 down-regulated genes. Notably, cross-comparisons identified only 135 common DEGs (LS vs. SS) both in the control and in darkness (Figure 4B), which possibly resulted from the traits of similar developmental processes. To explore the gene co-expression patterns of the DEGs in the comparison between LS and SS under light or dark conditions, 1797 DEGs were classified into eight clusters (Figure 4C). It was found that genes in C1 (304) and C2 (167) were down-regulated during DIS and showed higher expression levels in LS compared with SS (Supplementary Tables S1 and S2). Compared with SS, the genes in clusters C3 (75) and C4 (369) were also highly expressed in LS, but there was no significant change between light and dark conditions (Supplementary Tables S3 and S4). Conversely, genes in C5 (312) and C6 (119) were significantly up-regulated during DIS, with higher expression levels in SS than in LS (Supplementary Tables S5 and S6). Genes in C7 (257) were highly expressed in SS under both conditions, which is contrary to C3 (Supplementary Table S7). Moreover, the expression levels of genes in C8 (194) were also higher in SS under light conditions, but under dark conditions, these genes were slightly down-regulated in LS and sharply down-regulated in SS (Supplementary Table S8). The cell surface receptor signaling and energy metabolism (light harvesting in photosystem I, glycolytic and starch catabolic processes, lipid storage and pyruvate metabolic processes) were obviously affected in the C1–C4 clusters, within genes up-regulated in LS. Chlorophyll biosynthesis and the activities of oxidoreductase and hydrolase, as well as those related to the response to cytokinin, were also enriched in these clusters. Genes in the C5–C8 clusters were mainly up-regulated in SS, most of which were related to carbohydrate metabolic process, cell redox and lipid homeostasis, phytohormone-associated processes (JA-mediated signaling and negative regulation of auxin-mediated signaling pathways), cell wall organization, and leaf senescence (Figure 4D). Presumably, co-expression changes in these genes between LS and SS could reflect strong transcriptional effects and the involved functional processes of regulating leaf senescence in foxtail millet. 

### 2.3. Multiple Biological Processes and Pathways Are Involved in Regulating Senescence Progression in Foxtail Millet during DIS

**Figure 5:**
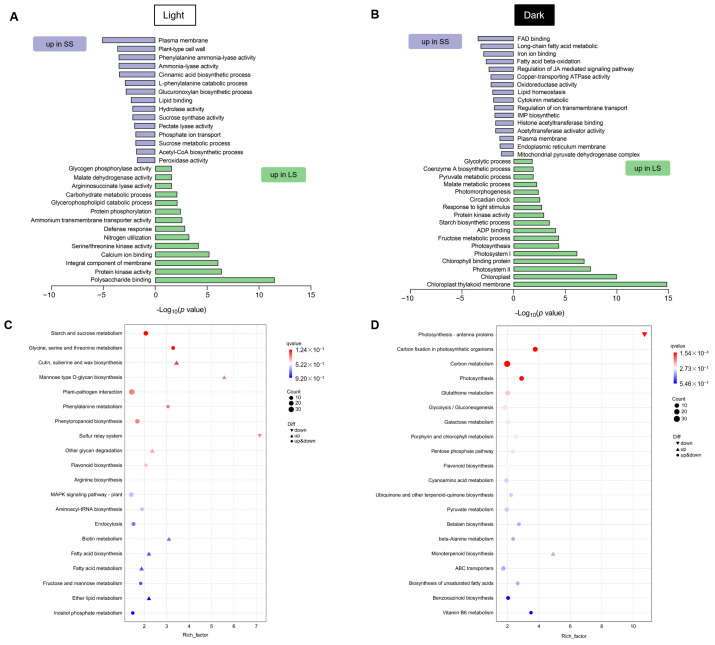
Specific GO term enrichment analysis for DEGs in the LS vs. SS group under (**A**) light and (**B**) dark conditions. *p*-value is the significance of the enriched GO terms. KEGG pathway enrichment for DEGs in the LS vs. SS group under (**C**) light and (**D**) dark conditions. Rich factor refers to the significant enrichment level of DEGs in this pathway.

To understand the molecular responses specific to the normal light or dark conditions in seedlings of foxtail millet varieties with different senescence characteristics, a further analysis was conducted on the 559 unique DEGs in light and the 1103 unique DEGs under darkness (Figure 4B). GO analysis of up-regulated DEGs in light-grown LS seedlings revealed that categories related to polysaccharide binding, integral membrane component, calcium ion binding, nitrogen utilization, defense response, protein phosphorylation, and the processes of carbohydrate metabolic and glycerophospholipid catabolic, as well as the activities of protein kinase, glycogen phosphorylase, malate dehydrogenase, argininosuccinate lyase, and ammonium transmembrane transporter, were significantly enriched. Conversely, GO categories up-regulated in light-grown SS seedlings included the activities of phenylalanine ammonia-lyase, ammonia-lyase, hydrolase, sucrose synthase, pectate lyase, and peroxidase and the processes of cinnamic acid biosynthetic, L-phenylalanine catabolic, glucuronoxylan biosynthetic, sucrose metabolic, and acetyl-CoA biosynthetic, as well as the plant-type cell wall, lipid binding, and phosphate ion transport (Figure 5A). 

Further, we analyzed DEGs under dark treatment by GO enrichment and found that categories associated with photosynthesis and chloroplast including chloroplast thylakoid membrane, chlorophyll binding protein, photosystem I and II, light stimulus response, and the processes of fructose metabolic, starch biosynthetic, malate metabolic, pyruvate metabolic, coenzyme A biosynthetic, glycolytic and circadian clock, etc., were significantly up-regulated in the LS. However, the GO categories up-regulated in the SS seedlings showed significant differences compared with LS, mainly including FAD binding, lipid homeostasis, ion (iron and copper) transmembrane transport, oxidoreductase activity, membranes (plasma and endoplasmic reticulum), hormones (JA and cytokinin) signaling and acetylization, etc. (Figure 5B).

Kyoto Encyclopedia of Genes and Genomes (KEGG) analyses revealed that genes associated with several aspects of amino acid and carbohydrate metabolism and phenylpropanoid and flavonoid biosynthesis as well as plant–pathogen interaction were enriched in the light-grown group of LS vs. SS. Further analysis found that categories related to lipid metabolism (fatty acid and wax biosynthesis), mannose type O-glycan biosynthesis, and other glycan degradation were significantly up-regulated in the SS, while the sulfur relay system was down-regulated (Figure 5C). The transcriptome changes caused by DIS revealed that genes associated with photosynthesis, carbon metabolism, and chlorophyll metabolism, as well as stress-related terms such as glutathione metabolism and flavonoid biosynthesis, were enriched. Further, the photosynthesis-antenna proteins were down-regulated in the SS, while the monoterpenoid biosynthesis was up-regulated (Figure 5D). 

### 2.4. Expression Changes in Senescence-Related Genes in Two Foxtail Millet Varieties

**Figure 6:**
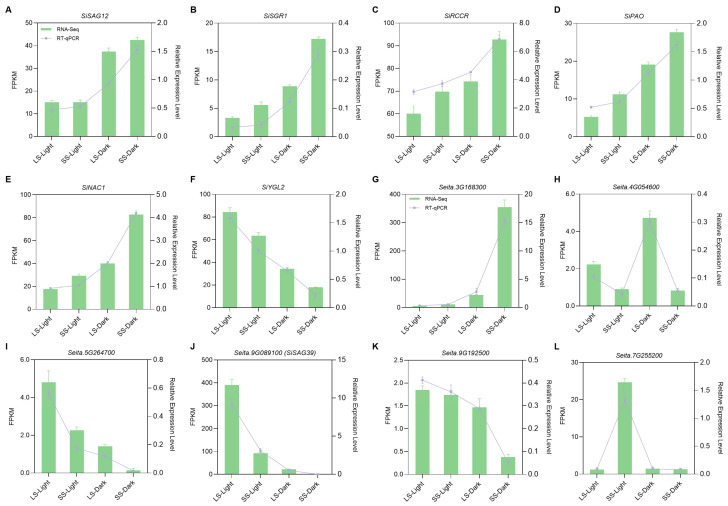
Expression profile of senescence-associated genes in two foxtail millet varieties during DIS. Transcriptome and quantitative real-time PCR analysis of (**A**) senescence hallmark gene; (**B**–**D**) chlorophyll-degradation-related genes; (**E**,**F**) positive and negative regulator of leaf senescence in foxtail millet; (**G**–**L**) *SiSAG*s selected from DEGs in LS and SS under light or dark conditions. Data are mean ± SD (*n* = 3 independent biological samples).

Physiological and biochemical changes during leaf senescence are influenced by the expression of *SAG*s. Here, we selected several *SAG*s according to previous studies and the functional annotation from the transcriptome data. As shown in Figure 6A, the hallmark senescence-progression-related gene *SiSAG12* was up-regulated by DIS in the two varieties, but the expression level was significantly higher in SS. The expression levels of genes associated with chlorophyll degradation, including *SiSGR1*, *SiRCCR*, and *SiPAO*, were consistently up-regulated after dark treatment. In addition, the expression of these genes was increased in SS both under light and dark conditions (Figure 6B–D). We also detected the expression of two other senescence-related genes, *SiNAC1* and *SiYGL2*, which serve as a positive regulator and a negative regulator, respectively, in the process of leaf senescence in foxtail millet [22,23]. In contrast, the expression of *SiNAC* was significantly up-regulated in SS compared to in LS, and the expression level of SiYGL2 was dramatically decreased, especially after dark treatment (Figure 6E,F). Next, we identified six *SiSAG*s from DEGs according to the functional annotation. It was found that the senescence regulator *Seita.3G168300* was induced by DIS, and the expression level was significantly higher in SS (Figure 6G). The flavonoid-biosynthesis-related gene *Seita.4G054600* showed a higher expression level in LS and was up-regulated after dark treatment but did not significantly change in SS (Figure 6H). The receptor protein kinase coding gene *Seita.5G264700* and the senescence-specific cysteine proteas coding gene *Seita.9G089100* (*SiSAG39*) were down-regulated by DIS in the two varieties, and their expression levels were approximately ten and twenty times lower in SS than in LS (Figure 6I,J). The receptor-like protein kinase (RLK) coding gene *Seita.9G192500* was significantly up-regulated in LS compared to SS after dark treatment, but the difference was not significant under light conditions (Figure 6K). The expression level of DNA catabolic-related gene *Seita.7G255200* was higher in SS than in LS under light conditions, but there was no difference between the two varieties after dark treatment (Figure 6L). RT-qPCR analysis was performed to validate the expression profiles of these *SAG*s in foxtail millet, and the expected expression pattern further confirmed the reliability of the transcriptome data.

### 2.5. Identification of Transcription Factors and Protein Kinases Related to the Regulation of Senescence Progression in Foxtail Millet

**Figure 7:**
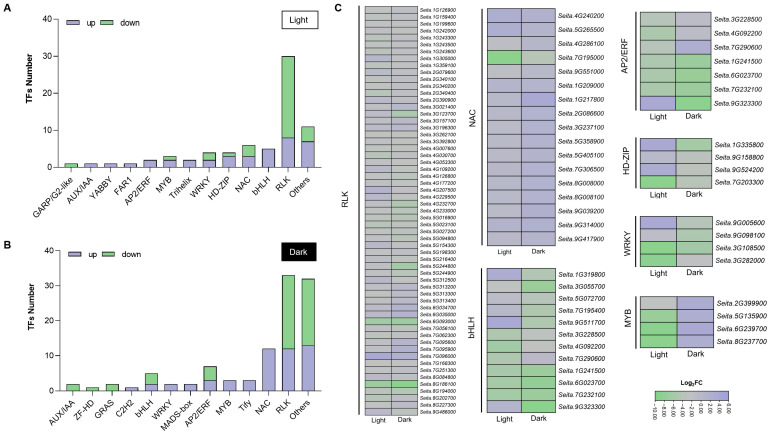
Analysis of TFs and PKs predicted from DEGs in the LS vs. SS comparisons under light or dark conditions. The number of (**A**) up- or (**B)** down-regulated differentially expressed TF or PK families; (**C**) a heat map showing the expression changes in genes encoding the main TFs or PKs. Each cell shows the log_2_ ratio of fold change in LS relative to that of SS under light or dark conditions.

Since TFs and protein kinases (PKs) play a key role in the regulation of plant senescence, we further identified eleven TF families and one PK family under light and dark conditions. As shown in Figure 7A, the typical representative TF families in the LS vs. SS group under light conditions included NAC (three up-/three down-regulated), bHLH (five up-regulated), HD-ZIP (three up-/one down-regulated), and WRKY (two up-/two down-regulated). Under dark conditions, NAC (twelve up-regulated), AP2/ERF (three up-/four down-regulated), bHLH (two up-/three down-regulated), and MYB (four up-regulated) were prominently over-represented in the comparison between LS and SS (Figure 7B). Significantly, most identified genes from the group of LS vs. SS belonged to the RLK family, including eight up-/twenty-two down-regulated and twelve up-/twenty-one down-regulated under light and dark conditions (Figure 7A–C). These RLKs may act as receptors to perceive extracellular signal molecules, including phytohormones and ROS, to regulate leaf senescence in foxtail millet. Although the most enriched TF families under the two conditions were similar and were widely reported in the regulation of leaf senescence, the expression patterns of several genes were obviously different (Figure 7C). The expression changes in these TFs suggested that they could have significantly different regulatory effects on *SAG*s in the DIS of the two foxtail millet varieties.

### 2.6. Screening for Modules and Candidate Genes Associated with Senescence Progression in Foxtail Millet Based on WGCNA

**Figure 8:**
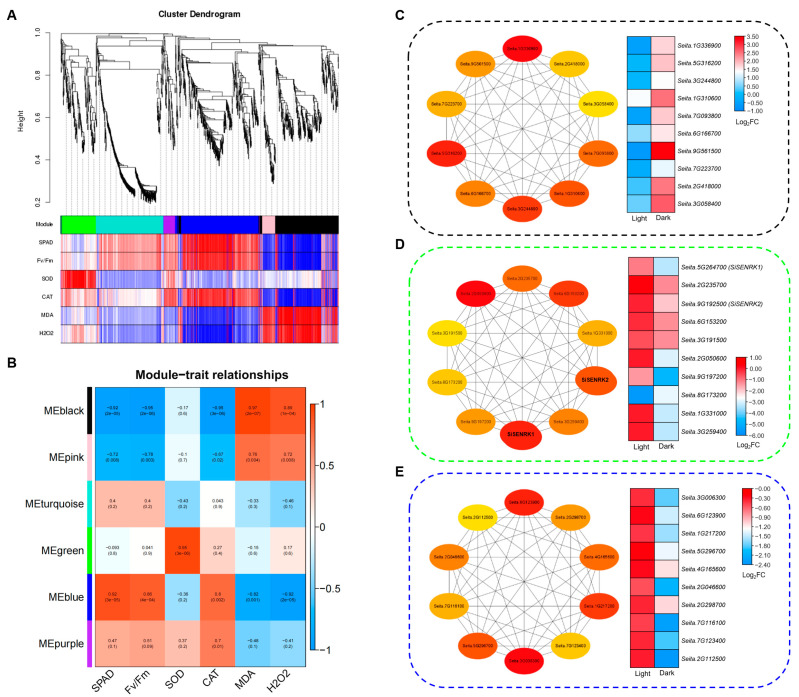
WGCNA analysis of DEGs involved in DIS of foxtail millet. (**A**) Hierarchical cluster tree of co-expression modules identified by WGCNA. The six major modules were labeled with different colors; (**B**) correlation analysis between gene modules and senescence-related traits; (**C**–**E**) top ten candidate hub genes selected from MEblack, MEgreen, and MEblue modules. Heatmaps showing the expression profiles of the hub genes from the representative modules.

To further identify the specific modules and genes associated with the senescence-related traits between LS and SS, we performed weighted gene co-expression network analysis (WGCNA) and obtained six distinct modules (Figure 8A). The relationships between module eigengenes and senescence-related physiological parameters indicated that the MEblack was positively correlated with the contents of H_2_O_2_ and MDA, whereas it was negatively correlated with values of SPAD and Fv/Fm. Conversely, the MEblue was positively correlated with values of SPAD and Fv/Fm as well as CAT activity but negatively correlated with the contents of H_2_O_2_ and MDA. Moreover, the MEgreen had a significant positive correlation with SOD activity (Figure 8B). These modules might have important biological roles in regulating senescence progression in foxtail millet. Hence, we performed KEGG pathway analyses of these important modules to reveal their potential functions. The results showed that genes in the MEblack were enriched mainly in the plant hormone signal transduction and metabolisms of carbohydrate and fatty acid (Supplementary Figure S1A). Additionally, the carbon metabolism, photosynthetic, and plant hormone signal transduction pathways were significantly enriched in the MEblue (Supplementary Figure S1B). However, the most enriched pathway in MEgreen was plant hormone signal transduction (Supplementary Figure S1C). Moreover, we screened the top ten hub genes among the three modules and further analyzed their expression changes. The results showed that the hub genes in MEblack were all up-regulated in SS compared to LS during DIS, whereas the hub genes in the MEblue and MEgreen were all down-regulated in SS (Figure 8C–E). The most significantly up-regulated gene in MEblack was related to flavonoid metabolism, *Seita.9G561500*, encoding a flavanone 3-dioxygenase (Figure 8C). Importantly, the senescence-related protein kinase (SENRK) coding genes *Seita.5G264700* (*SiSENRK1*) and *Seita.9G192500* (*SiSENRK2*) were also identified in MEgreen, which might negatively regulate leaf senescence in foxtail millet (Figure 8D). *Seita.7G123400* and *Seita.2G112500* were genes with the highest expression in the MEblue, which were related to carbohydrate transport and metabolism (Figure 8E).

## 3. Discussion

### 3.1. Physiological Characteristics of Foxtail Millet Exhibiting Varied Degrees of Senescence in Response to DIS

Leaf senescence is an intricate biological process encompassing multiple genetic elements and the integration of various environmental signals [2]. DIS is known to induce a rapid and synchronous senescence phenotype regardless of the developmental stage of plants with different genotypes [31,32]. Thus, we adopted this experimental system to investigate the physiological and biochemical changes in LS and SS foxtail millet seedlings during leaf senescence. The progressive loss of chlorophyll and reduction in photosynthesis are the hallmarks of leaf senescence [33]. Our data showed that the decreases in chlorophyll content and the maximum quantum efficiency of PSII photochemistry (Fv/Fm) elicited by DIS were significantly alleviated in LS compared to in SS, which was consistent with the senescence phenotype changes between the two varieties (Figure 1A–C). Furthermore, LS exhibited a sustained high photosynthetic capacity when compared to SS, and the rate of decline in photosynthetic parameters during DIS was slower (Figure 1D–G). Consequently, the delayed leaf senescence observed in LS is presumably caused by the inhibition of chlorophyll degradation, thereby enhancing photosynthetic ability. Excessive ROS production in dark-stressed plants causes membrane lipid peroxidation, leading to leaf senescence [34,35]. H_2_O_2_ and MDA levels were found to be lower in LS compared to SS during DIS, which could be explained by the increased activities of antioxidant enzymes (SOD and CAT) in LS (Figure 2). These results suggest that the activation of the enzymatic ROS scavenging system in LS reduced ROS accumulation and delayed its dark-induced leaf senescence, which was supported by previous studies in other plant species [26,36]. Flavonoids were previously reported to possess the capacity to scavenge ROS and mitigate oxidative damage in plants [37]. Similarly, our results indicated that LS exhibited a higher leaf flavonoid index, which might result in a reduction in ROS accumulation. Thus, it is likely that the variation in ROS scavenging capacity could account considerably for the differences in senescence phenotypes during DIS between LS and SS. The leaf senescence process in higher plants is modulated by internal hormone signals. Phytohormones, specifically ethylene, ABA, JA, and SA, are well-established inducers of leaf senescence, either through elevated endogenous levels or via exogenous application [38]. Auxin and cytokinin have been reported to serve as negative regulators of leaf senescence [6]. Our studies revealed that LS delayed senescence by suppressing the elevation of senescence-promoting hormones (ABA, JA, and SA) while mitigating the depletion of senescence-suppressing hormones (IAA and zeatin) (Figure 3). Intriguingly, ethylene serves as a widely recognized positive regulator of leaf senescence, yet there was no significant difference observed in the precursor ACC content between the two varieties. However, further research is needed to elucidate the underlying mechanism governing this hormonal regulation between LS and SS. Previous studies suggested that delaying leaf senescence successfully contributes to plant productivity and fitness [2], and therefore, the senescence-delaying varieties such as ‘LS’ could be useful for developing high-yield and stress-resistant varieties in foxtail millet.

### 3.2. Transcriptional Regulation of Dark-Induced Leaf Senescence in Foxtail Millet

Leaf senescence is a senescence process at the organ level that involves drastic changes in gene expression and metabolic processes. RNA sequencing has been successfully used to analyze the global gene expression profiles during leaf senescence to elucidate the molecular mechanisms [1,33,39]. Taking into account the fact that the DEGs between the two varieties could be due to different genetic backgrounds, we performed a comparative analysis of the global gene expression profiles between two foxtail millet varieties exhibiting significantly distinct senescence responses under both light and dark conditions. DEG clustering and GO enrichment categories suggested that genes associated with photosynthesis, chlorophyll biosynthesis, oxidoreductase activities, cytokinin response, and metabolic pathways such as lipid and starch were up-regulated in LS (Figure 4D), whereas clusters up-regulated in SS genes were mostly associated with redox homeostasis, carbohydrate metabolism, lipid homeostasis, hormone signaling, and leaf senescence. These gene expression patterns were similar to those in dark-induced or age-dependent leaf senescence from previous transcriptome data of other plants [24,39,40]. Significantly, the measured contents of phytohormones, including JA, IAA, and cytokinin, were in agreement with the DEGs involved in hormone signaling pathways. The redox regulation of NADP-malate dehydrogenase activity is crucial for maintaining NADPH homeostasis in chloroplasts and the optimal growth of plants during light–dark transitions and prolonged darkness [41]. Our transcriptome data showed that genes related to malate dehydrogenase activity and malate metabolic process were significantly up-regulated in the LS both under light and dark conditions (Figure 5A,B). It is therefore not unexpected that LS exhibits better resistance during DIS. Additionally, GO enrichment categories suggested that under normal light conditions, increases in transcripts associated with polysaccharide and calcium ion binding as well as nitrogen utilization and defense response were detected in LS, whereas genes involved in cellular components such as the plasma membrane and cell wall, as well as phenylalanine catabolic metabolism, were significantly up-regulated in SS (Figure 5A). These data might reflect the differences in developmental status and environmental adaptability between the two foxtail millet varieties. KEGG enrichment analysis further revealed that genes related to lipid metabolism, including fatty acid and wax, were significantly up-regulated in SS under light conditions (Figure 5C). We expected that this might be due to lipid homeostasis and turnover being disrupted in SS foxtail millet during the developmental process, resulting in an accelerated senescence phenotype in response to environmental stress [42,43]. Furthermore, we observed that genes up-regulated in LS during DIS were largely associated with photosynthesis and chlorophyll metabolism (Figure 5B,D), which is in line with the beginnings of leaf senescence and might explain the conspicuous difference in the chlorophyll content and photosynthetic capacity between LS and SS [44,45]. Secondary metabolites such as glutathione act as antioxidants to help plants scavenge ROS. Our transcriptome analysis revealed the enrichment of DEGs induced by DIS in the glutathione metabolite pathway, suggesting that glutathione plays a crucial role in regulating leaf senescence in both LS and SS through its antioxidant-enhancing capabilities. Taken together, the senescence patterns in foxtail millet seedlings during DIS were probably regulated by the integration of multiple biological processes and metabolic pathways. In particular, genes up-regulated in LS allowed us to identify the transcriptional regulation of the processes that may determine the delayed senescence phenotype and physiological changes in foxtail millet. It was also found that the hallmark *SAG*s and chlorophyll-degradation-related genes were significantly up-regulated in SS (Figure 6A–E), and many senescence-associated TF genes, such as *NAC*s and *WRKY*s [31], were greatly affected in the two foxtail millet varieties (Figure 7). Thus, it is likely that the regulation of the leaf senescence process in foxtail millet during DIS occurs at the transcriptional level of key *SAG*s via the TF regulatory cascade.

### 3.3. Candidate SAGs That Regulate Dark-Induced Leaf Senescence in Foxtail Millet

Previous studies have suggested that senescence-manipulating technology utilizing the functional genes that delayed leaf senescence could extend the photosynthetic period to increase the carbohydrates and improve yield in maize, rice, and wheat [24,46,47]. Therefore, identifying genes that postpone leaf senescence is a promising way to improve crop yields [18,46]. Here, we first identified a candidate *SAG* (*SiSAG39*) from DEGs that were down-regulated by DIS and had higher expression in LS than in SS (Figure 6J). It has been reported that the promoter of *SAG39* is implicated in the negative regulation of leaf senescence in rice [48]. A comparative transcriptome analysis further revealed that *TaSAG39* delayed the senescence process by maintaining the stability of chlorophyll–protein complexes in a stay-green wheat cultivar [26]. Our research also indicated that the senescence-delaying foxtail millet LS maintained a higher chlorophyll content during the DIS (Figure 1B). RLKs are an important class of plasma membrane regulatory proteins that enable plants to respond to various environmental stresses, which play a vital role in plant growth and development processes. Furthermore, several studies reported regulatory roles of RLKs in leaf senescence in rice and Arabidopsis [15,49], which negatively regulate dark-induced or age-dependent leaf senescence. Interestingly, our data showed that the expression of RLKs was most affected when comparing LS vs. SS under both light and dark conditions (Figure 7C), suggesting that RLKs have a strong effect on the progression of dark-induced leaf senescence in foxtail millet. Thus, we further screened two senescence-related RLK-encoding genes (*SiSENRK1* and *SiSENRK2*) based on WGCNA and RT-qPCR analysis. These two *SiSENRK*s were identified as hub genes in the MEgreen module, which exhibited a significant positive correlation with SOD activity. It was suggested that the expression of *SiSENRK1* and *SiSENRK2* was significantly down-regulated during dark-induced senescence and was highly expressed in LS (Figure 6I,K and Figure 8D). Based on prior studies and our results, it could be proposed that these *SAG*s might act as negative transcriptional regulators of dark-induced leaf senescence in foxtail millet, which requires further experiments to validate. 

**Figure 9:**
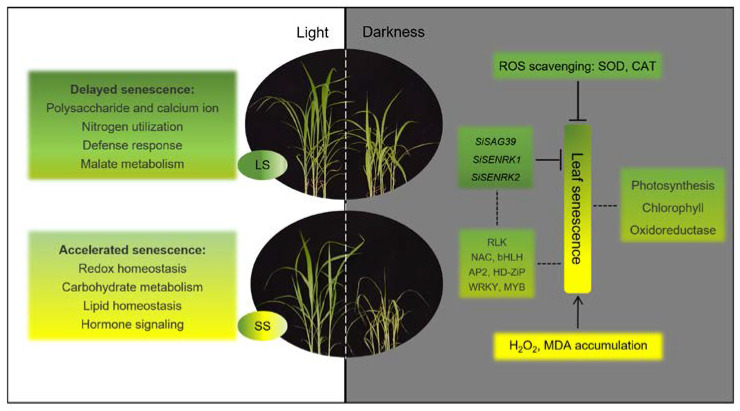
Proposed regulation model of dark-induced leaf senescence in foxtail millet.

In summary, we propose a model of the physiological changes and transcriptional regulatory mechanism underlying dark-induced leaf senescence progression (Figure 9). This study identifies several key biological processes and specific candidate genes that will help to further characterize the mechanisms regulating leaf senescence in foxtail millet.

## 4. Materials and Methods

### 4.1. Plant Materials and DIS Experiment

Two varieties of foxtail millet, B56 (LS) and B304 (SS), were selected from the Chinese core collection of foxtail millet germplasm resources using the DIS system. The Chinese core collection of foxtail millet germplasm resources was obtained from the Center for Crop Germplasm Resources of the Chinese Academy of Agricultural Sciences (Beijing, China). To grow the foxtail millet plants, surface-sterilized seeds were sowed in pots with nutrient soil under the photoperiod of 16 h light/8 h dark at 25 °C/28 °C for 4 weeks. For the DIS experiment, 4-week-old seedlings were transferred to complete darkness for 7 days, and the seedlings under normal photoperiod growth were used as the control group. Each treatment consisted of five pots with six seedlings in each pot, and three biological repetitions were performed. Fresh samples of leaves were immediately collected and frozen in liquid nitrogen, then stored at –80 °C for further analyses.

### 4.2. Measurement of Physiological and Biochemical Indicators

The chlorophyll content of leaves was measured with a chlorophyll meter, SPAD-502 Plus (Konica Minolta). The maximum photochemical efficiency of PSII, Fv/Fm, of leaves was measured with a chlorophyll fluorescence meter (WALZ MINI-PAM-II, Heinz-Walz Instruments, Effeltrich, Germany), as described previously [50]. Leaf gas exchange was measured on the leaves with the LI-6800 portable photosynthesis system (LI-COR Inc., Lincoln, NE, USA). Flavonoid content was determined using the Dualex 4 Scientific^+^ (Dualex Scientific, Force-A, Orsay-Cedex, France), through a quantitative comparison of light delivered to and emitted back from the leaf. Each leaf was measured at least ten times, and the data were represented by the average value. H_2_O_2_ content was measured with the titanium sulfate method using the Hydrogen Peroxide Content Assay Kit (Boxbio, AKAO009M, Beijing, Chian) according to the instructions, and DAB staining was performed using the DAB solution (Coolaber, SL1805, Beijing, China), as described previously [51]. MDA content was measured with thiobarbituric acid (TBA) colorimetry using the Malondialdehyde Content Assay Kit Assay Kit (Boxbio, AKFA013M). The activities of SOD and CAT were measured with the nitroblue tetrazolium (NBT) and the ammonium molybdate colorimetry methods using the Superoxide Dismutase Activity Assay Kit (Boxbio, AKAO001M) and the Catalase (CAT) Activity Assay Kit (Boxbio, AKAO003-2M), respectively, according to the instructions. Endogenous phytohormones including ABA, JA, SA, ACC, IAA, and zeatin were quantified using the HPLC/MS method, as described previously [52]. 

### 4.3. RNA Extraction and RT-qPCR Analysis

Total RNA was extracted from leaves of dark or light treatments using the FastPure^®^ Plant Total RNA Isolation Kit (Vazyme, RC401, Nanjing, China). First-strand cDNA was synthesized using the HiScript II Q RT SuperMix (Vazyme, R233), and the quantitative real-time PCR analysis was performed using the ChamQ Universal SYBR qPCR Master Mix (Vazyme, Q711) on the Bio-Rad Real-Time PCR System (Carlsbad, CA, USA). The relative expression level was calculated by the 2^−∆∆Ct^ method, and the *SiActin* was used for normalization. Primers used for RT-qPCR analysis are listed in Supplementary Table S9.

### 4.4. Transcriptome Profiling

RNA samples from the leaves of each treatment were used as input material, and RNA sequencing was performed on the Illumina high-throughput sequencing platform, which was conducted by Biomarker Technologies Co., Ltd. (Beijing, China). Raw data in fastq format were cleaned by removing reads containing adapter, reads containing ploy-N, and low-quality reads, and then the high-quality clean data were used for the downstream analyses. The numbers of counts per gene were normalized to fragments per kilobase of transcript per million mapped reads (FPKM) values before analysis of transcript abundance. DESeq2 was used to identify DEGs with a false discovery rate (FDR) < 0.01 and |Log_2_(FC)| > 1 [53]. GO and KEGG enrichment analyses of DEGs were performed using the GOseq R packages (https://bioconductor.org/packages/release/bioc/html/goseq.html, accessed on 5 September 2023) [54] and KOBAS software (http://kobas.cbi.pku.edu.cn/, accessed on 8 September 2023) [55]. TF/PK identification from DEGs was performed based on the BMKCloud (www.biocloud.net, accessed on 15 October 2023). The heatmaps were generated using TBtools (https://github.com/CJ-Chen/TBtools, accessed on 12 January 2024) [56].

### 4.5. WGCNA Analysis

We performed gene co-expression networks analysis of the transcriptome data using the WGCNA on the platform BMKCloud (www.biocloud.net, accessed on 16 November 2023). The correlation between gene modules and senescence-related traits was calculated by Pearson’s correlation coefficients, which is indicated by the depth of the color in the heatmap. The hub genes of the modules were selected and visualized using the Cytoscape (version 3.8.0) software.

### 4.6. Statistical Analysis

One-way ANOVA was used for statistical analysis in the LS and SS comparison under light or dark conditions. Data were the means ± SD of replicates from the measured parameters, and multiple comparisons were performed by Tukey’s honestly significant difference post hoc test at the 0.05 level. All data analyses were conducted based on at least three biological replicates. 

## 5. Conclusions

The senescence-delaying foxtail millet could maintain higher levels of Fv/Fm, chlorophyll content, and antioxidant capacity during DIS than the senescence-accelerating variety. Comparative transcriptome analysis revealed that the up-regulation of polysaccharide and calcium ion binding, nitrogen utilization, defense response, and malate metabolic-related genes might delay the leaf senescence process in foxtail millet during DIS. The gene expression changes related to redox homeostasis, carbohydrate metabolism, lipid homeostasis, and hormone signaling could accelerate dark-induced leaf senescence in foxtail millet. Additionally, *SiSAG39*, *SiSENRK1*, and *SiSENRK2* were identified as negative regulators of dark-induced leaf senescence in foxtail millet. This study uncovered the transcriptomic differences between foxtail millet exhibiting delayed and accelerated senescence phenotypes during DIS, thereby providing valuable insights into senescence resistance molecular breeding.

## Data Availability

The RNA sequencing data generated and analyzed during this study have been deposited in the National Center for Biotechnology Information (NCBI) Sequence Read Archive (SRA) database under the accession PRJNA1077864 (http://www.ncbi.nlm.nih.gov/sra/, accessed on 20 February 2024). Additional supplementary data for this study can be accessed in the online Supplementary Materials.

## Supplementary Materials

The following supporting information can be downloaded at https://www.mdpi.com/article/10.3390/ijms25073905/s1.
