# Supplementary figures and images for: Leaf Senescence Regulation Mechanism Based on Comparative Transcriptome Analysis in Foxtail Millet
Source: Int J Mol Sci. 2024 Mar 31;25(7):3905. doi: 10.3390/ijms25073905 (PMC11011800; doi:10.3390/ijms25073905)

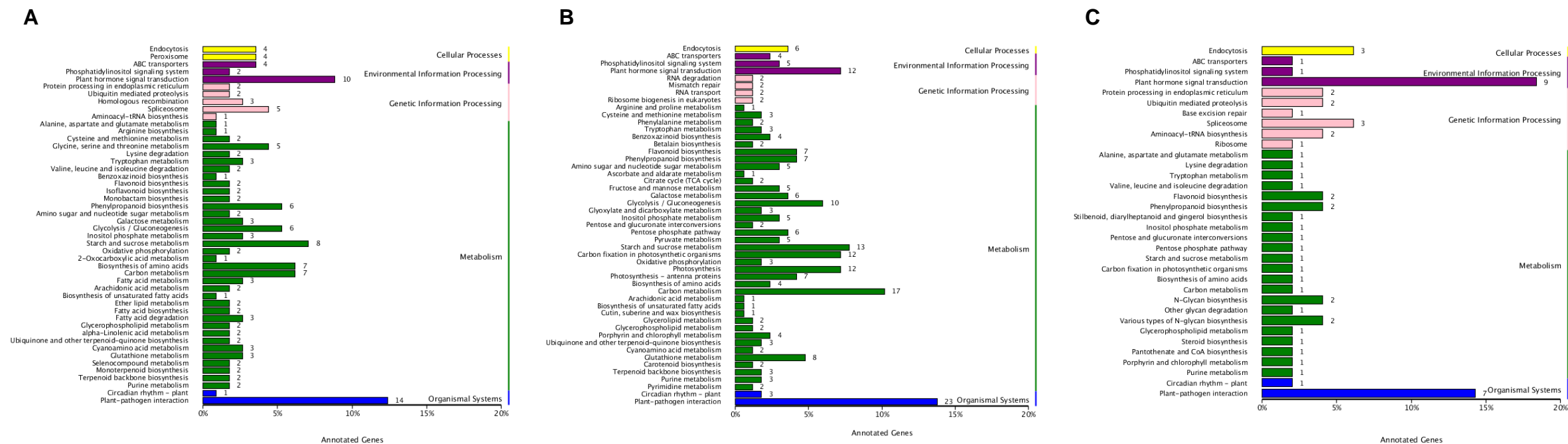

Figure S1. KEGG pathways enrichment for DEGs in (A) Meblack, (B) Meblue, and (C) Megreen.

Supplement: Supplementary file 1 [file ijms-25-03905-s001.zip › Supplementary Figure S1.pdf]
